# The COVID-19 pandemic as a starting point to accelerate improvements in health in our cities through better urban and transport planning

**DOI:** 10.1007/s11356-021-18364-8

**Published:** 2021-12-29

**Authors:** Mark J. Nieuwenhuijsen, Omar Hahad, Thomas Münzel

**Affiliations:** 1 ISGlobal, Barcelona, Spain; 2 Universitat Pompeu Fabra (UPF), Barcelona, Spain; 3 CIBER Epidemiología Y Salud Pública (CIBERESP), Madrid, Spain; 4 Mary MacKillop Institute for Health Research, Melbourne, Australia; 5 Department of Cardiology, Cardiology I, University Medical Center of the Johannes Gutenberg University Mainz, Langenbeckstrasse 1, 55131 Mainz, Germany; 6 German Center for Cardiovascular Research (DZHK), Partner Site Rhine-Main, Mainz, Germany; 7 Leibniz Institute for Resilience Research (LIR), Mainz, Germany

Cities are arguably the greatest achievement of human civilization. A greater proportion of the global human population is living in cities than ever, and this keeps growing with up to 70% of people expected to live in cities within 20 years. Jobs, wealth and prosperity, innovation, culture, entertainment, and access to healthcare are some of the greatest drivers of the growth and success of cities. But because of their density, cities also tend to be hotspots of air pollution and noise exposure, heat island effects, and more lately the COVID-19 pandemic (Nieuwenhuijsen 2020). Particularly, the more deprived were at higher risk of COVID-19 and already existing inequalities in cities were magnified. Cities also tend to have higher rates of poor mental health, and there were reports of further increases, particular during lockdowns (Kunzler et al. 2021).

During the COVID-19 pandemic, we have seen some dramatic changes to our cities with less use of public transport, fewer or almost no tourists, more teleworking, and even people moving out of the cities. Implemented lockdowns demonstrated what cities could achieve such as much less congestion and substantially lower noise and air pollution levels, particularly by decreases in particulate matter (PM) and nitrogen dioxide (NO_2_) (Munzel et al. 2021a, b). The reduction of air pollution levels led many cities to rethink their use of public space and transportation system, for example by reducing space for private motorized traffic and providing more infrastructure for active transport such as cycling and walking (Kraus and Koch 2021).

More than 8 million people each year die prematurely because of air pollution (Lelieveld et al. 2019), more than 3 million because of insufficient physical activity and more than 1 million because of traffic fatalities (Nieuwenhuijsen 2020). In addition, more than 120 million Europeans are exposed to noise levels above 55 dB(A), which were shown to increase the risk of cardiovascular disease (Munzel et al. 2021a, b). Furthermore, we are facing consequences of the climate crisis with stronger heat island effects in cities and growing population in Asia and Africa and aging population in Europe (Nieuwenhuijsen 2020).

We need to understand that the pre-COVID-19 pandemic urban and transport practices had a large impact on the physical and mental health in the population with related exposures in a city like Barcelona accounting for 20% of mortality premature, in addition to increased rates of cardiovascular and respiratory disease, cancer, diabetes, childhood asthma, and Alzheimer’s disease (Nieuwenhuijsen 2020). A substantial proportion of our cities are car-dominated but for different reasons when comparing various cities. Exemplarily, older European cities were originally built for walking and horse carriages, and particularly the city centers tend to be more compact than, for example, later North American and Australian cities, that were built for the car wither higher degrees of urban sprawl. European cities tend to have large public transport systems, while North American and Australian cities tend to rely on car-dominated mobility. The car density per square kilometer can still be higher in European cities than in North American and Australian cities, since there is less space available leading to, for example, higher air and noise pollution levels and related disease burden (Munzel et al. 2021a, b).

Although trends were noticed that people left the big cities during the early phase of the COVID-19 pandemic, more recent data indicates that relatively few people moved permanently (MCR 2021). Reasons for leaving the cities appear not to be the fear of infections, but, for example, loss of jobs or income and better opportunities to telework. Continuing telework as a constant feature may lead to reduced car-dominated travelling, to a better work-life balance and better quality of (family) life, and importantly reduced air and noise pollution levels (Munzel et al. 2021a, b).

However, people will continue to live in cities, and, therefore, there is a need to make our cities more sustainable, livable, and healthier and also more resilient with respect to future pandemic scenarios highlighting the need for systemic and multi-sectoral approaches. Technological changes to improve the transport sector like the introduction of electric cars have been implemented to address the climate crisis and to reduce air pollution, but they are hardly the solution to address the public health burden in its full magnitude (Nieuwenhuijsen 2020). Local tailpipe emissions and noise levels will be reduced, but the infrastructure and space for electric cars will take up as much as for fossil fuel cars and also electric car driving does not promote physical activity. Thus, we will need substantial changes to our urban design and lifestyle.

In many cities, we have seen during the COVID-19 pandemic that road space for motorized traffic has been reduced to increase the space for walking and cycling, leading to better opportunities for physical activities among children and the elderly. Furthermore, we observed that the availability of green spaces is an important resource as people craved nature. Herein, the quality of the urban green space is an important factor facilitating physical activity in older and the most susceptible populations. Numerous studies have demonstrated that increased physical activity is associated with access to and use of green space among senior citizen, working adults, and children. Importantly, the presence of trees in urban green spaces has been related with improvements in air quality due to trees capacity of removing pollutants from the atmosphere. Thus, increasing urban green space may result in a win–win situation related to increases in physical activity and improvements of air quality (Nieuwenhuijsen 2020; Munzel et al. 2021a, b).

To make a step towards better urban planning, the city of Barcelona has introduced the Superblock model, wherein motorized traffic is discouraged and active transportation and green space are encouraged (Bereitschaft and Scheller 2020). London introduced low-traffic neighborhoods, where motorized traffic is discouraged and people activities are encouraged. Paris is introducing the 15-min city, where all kind of destinations, work, school, shops, culture and leisure facilities should be reached within a 15-min walk or bike ride from home. Hamburg city is planning to be car-free by the year 2034 (Munzel et al. 2021a, b). These new urban models all aim to provide more public space for people and less space for individual motorized traffic and thereby to reduce air pollution and noise levels, greenhouse gas emissions, and heat island effects and increase physical activity and green space, which all improve the health of population (Nieuwenhuijsen 2020).

In order to achieve this, key actions will include to implement sufficient density, diversity (land-use mix) and destination accessibility, short distance to transit, desirability, walkability, and green space accessibility. For example, surrounding greenness, access to green space and visits to green space have been shown to increase life expectancy, improve cognitive functioning in children and the elderly, improve mental health, and stimulate the immune system (Nieuwenhuijsen 2020).

Drawbacks of the COVID-19 pandemic include the increase in online shopping leading to a decline of the local shopping infrastructure and increased local motorized traffic due to the deliveries. A vibrant and lively shopping and social infrastructure is essential for cities creating opportunities to meet people and to be socially active. Large inequalities existed in cities well before the COVID-19 pandemic, and these are becoming even more pronounced during the COVID-19 pandemic (Nieuwenhuijsen 2021).

The ecological/energy transition from fossil fuel to renewable energies that needs to be implemented in order to counteract the climate crisis will not only promote the sustainability and resilience of our cities, but also the livability and public health. The COVID-19 pandemic can be seen as crucial starting point to accelerate some of the developments that are urgently needed. The transition is costly in the short-term but cost-effective and essential in the long-term. The COVID-19-related financial stimulus packages such as the Biden’s Infrastructure Plan and the Next Generation EU fund can contribute to achieve improvements in urban and transport practices and provide an excellent opportunity to improve public health.

## Data Availability

Not applicable.
